# Investigation of outliers of evaluation scores among school of health instructors using outlier - determination indices

**Published:** 2016-01

**Authors:** HAMIDREZA TABATABAEE, FARIBA GHAHRAMANI, ALIREZA CHOOBINEH, MONA ARVINFAR

**Affiliations:** 1 Department of epidemiology, Shiraz University of Medical Sciences, Shiraz, Iran;; 2 Department of epidemiology, School of Health, Shiraz University of Medical Sciences, Shiraz, Iran;; 3 Research Center for Health Sciences, Shiraz University of Medical Sciences, Shiraz, Iran;; 4 School of Health, Shiraz University of Medical Sciences, Shiraz, Iran

**Keywords:** Outliers, Professors, Students, Evaluation

## Abstract

**Introduction:**

Teacher evaluation, as an important strategy for improving the quality of education, has been considered by universities and leads to a better understanding of the strengths and weaknesses of education. Analysis of instructors’ scores is one of the main fields of educational research. Since outliers affect analysis and interpretation of information processes both structurally and conceptually, understanding the methods of detecting outliers in collected data can be helpful for scholars, data analysts, and researchers. The present study aimed to present and compare the available techniques for detecting outliers.

**Methods:**

In this cross-sectional study, the statistical population included the evaluation forms of instructors completed by the students of Shiraz School of Health in the first and second semesters of the academic year 2012-2013. All the forms related to these years (N=1317) were entered into analysis through census. Then, four methods (Dixon, Gauss, Grubb, and Graphical methods) were used for determining outliers. Kappa coefficient was also used to determine the agreement among the methods.

**Results:**

In this study 1317 forms were completed by 203 undergraduate and 1114 postgraduate students. The mean scores given by undergraduates and postgraduates were 17.24±3.04 and 18.90±1.82, respectively. The results showed that Dixon and Grubb were the most appropriate methods to determine the outliers of evaluation scores in small samples, because they had appropriate agreement. On the other hand, NPP and QQ plot were the most appropriate methods in large samples.

**Conclusion:**

The results showed that each of the studied methods could help us, in some way, determine outliers. Researchers and analysts who intend to select and use the methods must first review the observations with the help of descriptive information and overview of the distribution. Determination of outliers is important in evaluation of instructors, because by determining the outliers and removing the data that might have been recorded incorrectly, more accurate and reliable results can be obtained.

## Introduction

Faculty member are important members of the educational system and need good circumstances to accomplish their required responsibilities. Therefore, performance evaluation and incentive measures are necessary to create motivation and improve the quality of education for students who are the future of the country (1). Educational evaluation has considerable applications in improving the quality of educational systems. Thus, teacher assessment is known as the most complex type of evaluation in any educational activity. This complexity results from low credibility of the used instruments and measurement methods (2).

Evaluation has been defined as gathering information about the activities, characteristics, and outcomes of programs to make judgments, improve the effectiveness of programs, or provide information for making future decisions (3). Teacher evaluation is considered as an important strategy for improving educational quality by universities and leads to better understanding of the strengths and weaknesses of training. However, the results of evaluation can be reliable when the collected information is accurate (4). Analysis of teacher evaluation scores in universities is one of the main fields of educational research (5). In fact, evaluation of teachers by students is one of the most important evaluations in the educational system and an important tool for teachers and educational administrators in educational planning (6). Although a lot of researches have been performed in this regard, most of the results are flawed due to the heterogeneity of the data (7).

In general, evaluation of faculty members is done through several models, including evaluation by administrators, colleagues, students, and self-assessment. Among these models, evaluation by students is most common in universities and educational institutions (8).

Given the many challenges in relation to student ratings in the recent years, lots of researches were carried out inside and outside the country. These studies have investigated various aspects of the results of such evaluations and have tried to identify their other dimensions by examination of different aspects and evaluation of documents. An extensive review article on student evaluations showed that the reliability of the evaluation results depended on the number of students. Accordingly, when the numbers of students in a class was more than 30, the results would be more reliable (9).

Outliers in evaluation scores are one of the problems that lead to wrong conclusions, which can be indicative of wrong measurement or recording. Therefore, identification of outliers is necessary in order to reach a correct conclusion.

An outlier is defined as an observation that "appears" to be inconsistent with other observations in the data set (10). An outlier is usually larger or smaller than the other values in a data set. Outliers occur for one of the following reasons:

1- Incorrect observed, recorded, or entered measurement in the computer,
2- Collection of measurements from different communities,
3- Measurements for expression of a rare accident or incident, and
4- Skewedness of most data in the frequency distribution curve (11).

In a previous study, critical values were determined for Dixon test (12). Outliers are effective in all stages of analysis and interpretation of information. In some cases, even there is no possible logical conclusion from the data set, resulting in statistical mistakes in terms of reliability and validity. Therefore, familiarity with methods for identification of outliers in the collected data can be useful for scholars, data analysts, and researchers (13). In fact, outliers can be used to identify strong and weak teachers and perform the necessary encouragements and notifications.

Up to now, no studies have been conducted on determination of outliers of teacher evaluation scores. Hence, the present study aims to present the techniques for identification of outliers and compare these methods regarding the results of evaluation of the quality of teaching. The researchers made use of Dixon, Gauss, Grubb, and Graphical methods for assessment (10).

## Methods

In the present cross-sectional research, the statistical population included the evaluation forms of instructors completed by the students of Shiraz School of Health. All the questionnaires were analyzed in the first and second semesters of the academic year 2012-2013. Sampling was done using census method.

At the time of the study, School of Health had 8 departments, including epidemiology, public health, environmental health, occupational health, health education, nutrition, medical entomology, and ergonomic. Some of these departments only had undergraduate students (public health), some of them only had postgraduate students (health education, medical entomology, ergonomics, epidemiology, and nutrition), and others had both undergraduate and postgraduate students.

The forms consisted of 15 questions related to undergraduate students (form3) and 17 questions related to postgraduate students (form9). In this study, 4 methods (Dixon, Gauss, Grubb, and Graphical) were used for determination of outliers.

**1-Dixon:** Dixon devised eight formulas for populations with different sizes with normal distribution. In this method, the maximum number is 30. Using this method, only one individual can be specified on each side of distribution (top and bottom) (Table 1).

**Table1:** Calculation of Dixon method

**Row **	**Number of people**	**Min**	**Max**
1	3-7	$\tau =\frac{x_{2}-x_{1}}{x_{n}-x_{1}}$	$\tau =\frac{x_{n}-x_{n-1}}{x_{n}-x_{1}}$
2	8-10	$\tau =\frac{x_{2}-x_{1}}{x_{n-1}-x_{1}}$	$\tau =\frac{x_{n}-x_{n-1}}{x_{n}-x_{2}}$
3	11-13	$\tau =\frac{x_{3}-x_{1}}{x_{n-1}-x_{1}}$	$\tau =\frac{x_{n}-x_{n-2}}{x_{n}-x_{2}}$
4	14-30	$\tau =\frac{x_{3}-x_{1}}{x_{n-2}-x_{1}}$	$\tau =\frac{x_{n}-x_{n-2}}{x_{n}-x_{3}}$

**2- Gauss:** This method is known as J. Gauss test and is done using the following formula:

$g=\frac{x_{exrt}-\overset{-}{x}}{s}$

The result of this formula was compared to the “g” value obtained from the Gauss table. If the obtained value was greater than the number in the Gauss table, it was considered as an outlier. This test is appropriate for populations of 3 to 50 people.

**3- Grubb:** This test is done based on the difference between the average and the end value by taking the standard deviation. Its formula is as follows:

$T_{min}=\frac{x_{mean}-X_{1}}{s}$

$T_{max}=\frac{x_{n}-X_{mean}}{s}$

After calculation, the low and high values were compared to the Grubb table. The obtained value was considered to be significant in case it was greater than the number in the table.

**4- Graphical methods:** Graphical methods are varied and have various functions. Among these methods, Quantile-Quantile plot, Box plot, and Normal Probability Plot (NPP) were used in the present study.

## Results

This study was conducted on 1317 forms completed by undergraduate, MSc, and Ph.D students. The mean scores of teacher evaluations given by undergraduate and postgraduate students were 17.24±3.04 SD and 18.9±1.82 SD, respectively.

1- Form3 (Undergraduate students) according to departments:

Outliers were 3.2% by Gauss method, 2.2% by Grubb method, 3.7% by Dixon method, and 15.3% by Graphical method.

2- Form3 according to fields of education:

Because the sample size was more than 30, Gauss, Grubb and Dixon methods could not be used for determination of outliers. Outliers were 15% according to Graphical method.

3- Form9 (postgraduate students) according to departments:

Outliers were 1% by Gauss method, 3% by Grubb method, 4% by Dixon method, and 41% by Graphical method.

4- Form9 according to fields of education:

Outliers were 0.8% by Gauss method, 3.6% by Grubb method, 5.4% by Dixon method, and 85% by Graphical method.

5- Form9 according to education levels:

Outliers were 0.5% by Grubb method, 0.5% by Dixon method, and 65.5% by Graphical method.

This study also examined the agreement between different methods for detecting outliers. The results indicated a significant agreement between Dixon and Gauss methods, Gauss and Grubb methods, and QQ plot and NPP in form No. 3 (undergraduate students) related to the departments. A significant agreement was also observed between QQ plot and NPP based on the field of education (Table 2).

**Table 2:** Agreement between methods for determining outliers in form3 (Undergraduate students)

	**Agreement**	**Kappa**	** CI **
Departments	Dixon and Gauss	0.65	0.434 - 0.868
	Gauss and Grubb	0.66	0.63 - 0.69
	QQ plot and NPP	0.61	0.55 - 0.645
Fields of education	QQ plot and NPP	0.68	0.67 - 0.69

There was a significant agreement between Dixon and Grubb methods in form9 (postgraduate students) related to the departments. A significant agreement was also found between Grubb and Box plot in this form related to the field of education. Additionally, a significant agreement was detected between QQ plot and NPP as well as between Grubb and Dixon methods based on education levels (Table 3).

**Table3:** Agreement between outlier-determining methods in form 9 (Postgraduate students)

Departments	**Agreement**	**Kappa**	**CI**
	Dixon and Grubb	0.65	0.55 – 0.75
Field of education	Grubb and Box plot	0.55	0.36 – 0.75
Educational levels	Dixon and Grubb	1	0.35 – 1.65
	QQ plot and NPP	0.94	0.83 – 1.06

## Discussion

The purpose of evaluating faculty members at universities is resolving deficiencies in teaching methods and complete and accurate transfer of knowledge from instructors to students. If this is done based on scientific criteria, the results can strengthen teaching, eliminate weaknesses, and provide a basis for decision-making and educational planning (14).

Determination of outliers is quite important in evaluation of instructors to remove the incorrectly recorded items and leads to achievement of more accurate and reliable results. In general, several tests are used to determine outliers. However, a previous study introduced Dixon test as the simplest and easiest method (15).

In this study, 1317 forms (forms 3 and 9) were investigated regardless of the professors’ names. The results showed the highest agreements between Dixon and Grubb methods and between QQ plot and NPP with kappa coefficients of 1 and 0.8, respectively. These agreements were shown in all the three groups (department, fields of education, and education level).

Moreover, Dixon and Grubb methods were most appropriate for determination of outliers of evaluation scores in small samples, because they showed suitable agreement. On the other hand, NPP and QQ plot were most suitable for large samples. Overall, the results showed that each of the studied methods could help us, in some way, to determine outliers. Researchers and analysts who are responsible for selecting and using the methods must first review the observations with the help of descriptive information and overview of the distribution. Then, they should use the appropriate method to determine and remove outliers. A previous study suggested using Grubb method with graphical methods and quarters (13).

With the help of outliers, teachers can be grouped into three categories (High, Middle and Low scores. 

## Conclusion

The individuals who are identified as outliers should be taken into account. Then, the reasons for their isolation must be determined and the related problems should be eliminated. The first group should be examined more closely and can provide a model for others. Finally, teachers are recommended to be divided into three groups based on the outliers and be compared in terms of personal and academic characteristics. Institutions that can benefit from the results of this research include Shiraz University of Medical Sciences, other training centers of the Ministry of Science, Islamic Azad University, and Payam-e-Noor University. 
